# Supplementary material for: Preliminary Study of the Antimicrobial, Anticoagulant, Antioxidant, Cytotoxic, and Anti-Inflammatory Activity of Five Selected Plants with Therapeutic Application in Dentistry
Source: Int J Environ Res Public Health. 2022 Jun 28;19(13):7927. doi: 10.3390/ijerph19137927 (PMC9265615; doi:10.3390/ijerph19137927)
Supplement: Supplementary file 1 [file ijerph-19-07927-s001.zip › COMITÉ BIOÉTICA FOLIO (00152).pdf]

SPSI – 010613
Folio: 00152

COMITÉ DE BIOETICA

Comisión de Revisión Bioética

Para:	Dra. en C. Myriam Angélica de la Garza Ramos
Fecha de recepción:	Noviembre del 2017
Título:	Evaluación de extractos vegetales como potencial alternativa en el tratamiento terapéutico pulpar aplicado a modelo animal
Para:	Sonia Martha López Villarreal

El presente es referencia para protocolo de investigación mencionado en la parte superior.
En la deliberación de este comité, los procedimientos en dicha aplicación conformada por las reglas y el reglamento del DHHS y de la FDA en relación con los temas de uso humano. La aprobación se otorga durante un año.
Como condición para aprobar la investigación, el responsable de la investigación debe de haber leído, establecido y firmado el escrito adjunto de nuestro Documento Federal de Seguridad.
Además el responsable de la investigación acuerda lo siguiente:

1. A dar información mediante un reporte de revisión periódica, necesaria para la revisión de este protocolo por parte del comité, en intervalos apropiados para evitar el riesgo y asegurar que el protocolo está siendo guiado con las recomendaciones y la supervisión del comité, pero dichos intervalos no deben tener más de un año desde su inicio.

2. Proveer al comité la forma del reporte periódico de revisión, así como el reporte final cuando concluya su proyecto.

3. El uso como documento de consentimiento informado para este estudio, el reporte final aprobado por el comité IRB impreso definiendo su periodo de aprobación.

4. Reportar cualquier evento adverso relacionado con el estudio y que pudiera afectar la salud mental y física del paciente.

5. Este estudio está sujeto a registro durante este periodo de tiempo.

Los registros relacionados con las acciones del comité referentes a este protocolo están en el archivo en la oficina de División de Estudios de Posgrado y de Investigación de esta Facultad.

Fecha de aprobación:	Noviembre del 2017
Periodo aprobado:	Noviembre del 2017 a Noviembre del 2018
Responsable de la Investigación	

Comité Institucional Investigador

Dr. Jaime Adrián Mendoza Tijerina

Dr. Roberto Carrillo González

Dra. Myriam de la Garza Ramos

La siguiente información describe las responsabilidades tomadas del Documento de archivo de Múltiple Seguridad, de la Universidad Autónoma de Nuevo León, Facultad de Odontología, junto con la Ley Federal de Salud en Materia de Investigación para la Salud (SS, 1987) ANEXO I.
La aprobación del protocolo está sujeta a estas reglas:

a) Los investigadores declaran y aceptan su responsabilidad para la protección de los derechos y el bienestar del humano así como garantizar su integridad.

b) Los investigadores que intenten involucrar investigaciones con humanos no estarán exentos de la aplicación de nuestras leyes federales y universitarias.

c) Los investigadores son responsables de otorgar una copia de la aprobación del IRB firmada, y el documento de consentimiento de cada periodo de tiempo a menos que el IRB elimine este requerimiento. Todos los documentos de consentimiento firmados serna guardados en la oficina administrativa de investigación.

d) Los investigadores reportaran con rapidez los cambios propuestos en las actividades de investigación relacionados con humanos al IRB.

e) Los investigadores son responsables para reportar el progreso de la investigación a la oficina administrativa de investigación, tan a menudo como se describe en las bases de riesgos del IRB, pero no menos de una vez al año.

f) Los investigadores deberán reportar inmediatamente los daños y/o problemas que sean riesgo para los sujetos y para otros miembros de la comunidad.

g) Ni el investigador, ni asociados interinstitucionales, no institucionales en acuerdos de investigación podrán buscar para su beneficio obtención de créditos, la utilización de la información de las intervenciones con el paciente que constituyan una violación a las garantías de su atención médica, sin la aprobación del IRB. Un médico deberá proveer seguridad ética/medica al paciente con la revisión y aprobación del IRB, exigido por la ley.

h) Los investigadores deberán notificar al IRB, a la oficina de Investigación Administrativa y a las instituciones oficiales el intento para la admisión de material humano que vayan a ser utilizados en los protocolos de investigación.

Capítulo 1, capítulo 13. Prevalecerá el criterio de respeto a su dignidad y la protección de sus derechos y bienestar, por lo cual se solicitara a los pacientes su aprobación voluntaria.

Artículo 14. Fracción V. Se contara con el consentimiento informado y por escrito del sujeto de investigación, en este caso se solicitara el consentimiento informado del paciente previo a la aplicación de los instrumento.

Fracción VI. Todos los estudios serán realizados por profesionales de la salud con conocimiento y experiencia para cuidar la integridad del ser humano, bajo la responsabilidad de una institución de atención a la salud.

Fracción VII y VIII. Se contara con el dictamen favorable de la Comisión de Investigación y Bioética de la Facultad de Odontología de la Universidad Autónoma de Nuevo León; la colecta de los datos se realizara solo cuando se cuente con dicha autorización.

Artículo 16. Se protegerá la privacidad del sujeto investigado, ya que no se solicitara identificación.

Artículo 17. Fracción I Esta investigación (es) debe de considerarse como riesgo mínimo.

Articulo 18 y 21. Para considerar existente el consentimiento informado del sujeto de investigación recibirá una explicación clara y completa de lo siguiente:

1) Justificación de los objetivos de investigación.

2) Los procedimientos que vayan a usarse y su propósito, incluyendo la identificación de los procedimientos que son experimentales.
3) Las molestias o riesgos esperados.
4) Los beneficios que pueda obtener.
5) Los procedimientos alternativos que pudieran ser verificados por el sujeto.
6) La garantía de recibir respuesta a cualquier pregunta y aclaración de los procedimientos, riesgos, beneficios y otros asuntos relacionados con al investigación en el tratamiento del sujeto.
7) La libertad de retirar su consentimiento en cualquier momento y dejar de participar en el estudio, sin que por ello creen perjuicios para continuar su cuidado y tratamiento.
8) La seguridad de que no se identificara al sujeto y se mantendrá la confidencialidad de la información relacionada con su privacidad.

He leído y comprendido mis responsabilidades antes descritas.

Firma del Investigador Principal
