# Supplementary material for: Preliminary Study of the Antimicrobial, Anticoagulant, Antioxidant, Cytotoxic, and Anti-Inflammatory Activity of Five Selected Plants with Therapeutic Application in Dentistry
Source: Int J Environ Res Public Health. 2022 Jun 28;19(13):7927. doi: 10.3390/ijerph19137927 (PMC9265615; doi:10.3390/ijerph19137927)
Supplement: Supplementary file 1 [file ijerph-19-07927-s001.zip › CONSENTIMIENTO INFORMADO UANL.pdf]

UNIVERSIDAD AUTONOMA DE NUEVO LEON
FACULTAD DE ODONTOOGIA
Dr. Eduardo Aguirre Pequeño y Silao s/n Col. Mitras Centro Monterrey N.L

CLINICA / MODULO:____

CONSENTIMIENTO INFORMADO

Yo ____ con fecha de nacimiento ___ de ____________ de _________ con folio de expediente ___________manifiesto que el Doctor(a) y/o estudiante de Odontología (o especialidad del área Odontológica) ______________________________________ me ha informado que ***es recomendable se me realice*** el siguiente **acto bucodental***:____

____

____y que seré atendido por el Doctor(a) y/o estudiante de Odontología (o especialidad del área Odontológica) ____

La(s) **alternativa(s) de tratamiento** es (son) la(s) siguiente(s): ____
____
____

He decidido realizarme el acto bucodental ____ de las opciones mencionadas anteriormente puesto que mis **motivos de elección** son: ______________________________
____

a pesar que se me han explicado *riesgos y beneficios* del procedimiento que acepto se realice y de las alternativas mencionadas a continuación

Dado el(los) siguiente(s) **diagnostico(s)**: ____
____
____

Con **pronóstico:** Bueno: ☐ Malo: ☐ Reservado: ☐
____
____

Categorizándolo como un acto odontológico: Urgente: ☐ De riesgo: ☐ No urgente: ☐
Otro (especificar): ☐
____
____

Se me infirmó que dadas mis **condiciones de salud general** se pueden presentar los siguientes riesgos antes, durante y/o después de realizado el acto bucodental: ____
____
____
____

De acuerdo a mi estado de Salud general se me considera como un paciente con **condiciones pre-anestésicas tipo** ☐
según los lineamientos señalados en la *NOM006 SSA3 2011 Para la práctica de anestesiología.*
Los **beneficios** de realizarme el acto odontológico señalado en este documento son: __________________________
____
____

Riesgo(s) del acto(s) sugerido(s): ____
____
____
____

El **procedimiento consiste en (descripción breve)**: ____
____
____
____
____

El acto odontológico generalmente se realiza con anestesia local en una o más citas de larga duración dependiendo de la situación bucodental, se utilizan materiales dentales con diversos componentes químicos, lo que puede tener complicaciones con la administración o contacto directo/continuado, como: alergia leve (edema, prurito, rash cutáneo, edema angioneurótico, irritación) hasta grave como shock anafiláctico (que amerite intervención de urgencia), taquicardia, parestesia temporal de la región infiltrada (por no más de 4 horas, por lo que se recomienda no hablar o comer en este lapso), limitación de apertura bucal, dolor en el sitio de la punción, hematomas, aftas, mareo.

Posibles Complicaciones o molestias del acto descrito:
Todas las mencionadas en el párrafo anterior (producidas por la aplicación de anestesia y química de los materiales dentales)
Limitación de apertura bucal o dislocación del maxilar inferior por la apertura bucal prolongada.

***Acto bucodental:** *Procedimiento, médico o quirúrgico con fines diagnósticos, terapéuticos, rehabilitatorios, paliativos o de investigación. Debe actualizar Consentimiento cada vez que modifique el plan de tratamiento*

Eventos que requieren consentimiento informado: **Procedimientos que requieren anestesia general o regional, Investigación clínica en seres humanos, Cualquier procedimiento que extrañe mutilación. Cada acto o cambio de plan de tratamiento (procedimiento/acto) genera un Consentimiento Informado.**

En ocasiones se pueden llegar a necesitar actos adicionales que pudieran significar un costo y riesgos adicionales a los descritos en este documento, mismos que de suscitarse se me harán saber.
El tratamiento propuesto tiene el(los) siguiente(s) **efecto(s) secundario(s):** alergia (inmediata, edema angioneurótico, dermatitis, rash cutáneo), irritación de las mucosas (por alergia o quemadura leve a los componentes de los materiales dentales), sensibilidad posoperatoria, dolor posoperatorio, ____
____
____
____

Al terminar el acto odontológico motivo de este Consentimiento se me ha comentado que es necesario con el fin de prolongar los beneficios del acto realizado realice las siguientes **recomendaciones posteriores al acto** descrito en este documento: ____
____
____
____
____

DECLARO que he COMPRENDIDO Y SE ME HAN ACLARADO TODAS MIS DUDAS de todo lo escrito en este documento y se me ha comentado que puedo *en todo momento aclarar las dudas que surjan antes, durante y después del tratamiento*. Estoy enterado(a) que es mi derecho, en todo momento ***confirmar o revocar*** la continuidad del acto odontológico que hoy asiento.
Reconozco que de no acudir a las citas en las fechas indicadas, el plan de tratamiento y costos puede modificarse ya que algunos padecimientos bucodentales pueden cambiar (avanzan) constantemente con el paso del tiempo.
Entiendo que la Facultad de Odontología de la U.A.N.L es una institución docente, asistencial, y de investigación. Por lo que de acuerdo a la ***NOM 004 SSA3 2012 Del expediente clínico*** comprendo que todos los estudios de imagen (Radiografías, fotografías, entre otros), laboratorio y gabinete, modelos de estudio, recibos de pago (a excepción del comprobante que me corresponde) necesarios para la atención permanecerán resguardados en el expediente clínico de esta institución, así como la Historia Clínica (que contiene: Diagnóstico, Pronóstico, Plan de Tratamiento, Notas de Evolución y Referencias) y que el personal que labora en este Centro Educativo está comprometido en respetar **mi derecho de titularidad y confidencialidad*** por lo que **la autenticidad y actualización de los datos proporcionados es responsabilidad mía como de quien la recaba.**
Sin embargo como institución de educación e investigación, comprendo que la información recopilada en la Historia Clínica y anexos pudiera ser utilizados con fines académicos y de divulgación*; Por lo cual manifiesto mi autorización de presentar salvaguardando mi identidad (*especificar*), la información y/o imágenes divulgadas en los diversos foros (académico-científicos y/o a la población general) **Autorizo la divulgación de información:** ☐ **SI** ☐ **NO**

Pueden mostrar: Rostro completo: ☐ Mostrar rostro con franja en los ojos: ☐ Tercio inferior de la cara: ☐
Solo el área de interés: ☐ Otro (especificar): ☐ ______________________________
Observación realizada por el paciente para su autorización: ______________________________
____

Nota: Manifiesto que de ser necesario realizar un acto de urgencia o contingencia derivada del acto autorizado, consiento al personal para realizar la intervención, ya sea odontoestomatológica y/o de urgencia que amerite de Resucitación Cardiopulmonar o cualquiera necesario para mi estabilización y resguardo de mi vida e integridad general o la de algún órgano (dentario o tejido blando bucal). En caso de *revocar mi autorización u abandonar el tratamiento*, deberá expedirse un registro de **Egreso Voluntario** (incluirá un resumen clínico) mismo que quedara asentado en la nota de evolución clínica.

***Aviso de Privacidad Art. 1, 2 párrafo quinto, 34, 35 fracción I, 43, 46, 47 y 51 de la Ley de Transparencia y Acceso a la Información del Estado de Nuevo León.**

____________________________ Nuevo León; a _______ de ______________________20___

____
Paciente/Padre, Tutor o familiar más cercano
Nombre completo y Firma de enterado y comprensión previa a la autorización de todo lo asentado en este documento
ID oficial con fotografía: Elector___ Licencia___ Cartilla militar____ Pasaporte____ Otro: _______________
FOLIO/NUMERO:____________________________

____
Estomatólogo Tratante
Nombre completo y Firma de haber informado y resuelto las dudas del motivo del Consentimiento Informado, previamente a la firma del asentamiento y acto odontológico.
Cedula Profesional/Matricula______________________

____
Testigo 1
Nombre completo y Firma de enterado
ID oficial con fotografía: Elector___ Licencia___ Cartilla militar____ Pasaporte____ Otro: _______________
FOLIO/NUMERO:____________________________

____
Testigo 2
Nombre completo y Firma de enterado
ID oficial con fotografía: Elector___ Licencia___ Cartilla militar____ Pasaporte____ Otro: _______________
FOLIO/NUMERO:____________________________

NOM 006 SSA3 2011 Para la Práctica de Anestesiología.
9. Lineamientos para el cuidado pre-anestésico
9.2 [enlista VII tipos] Preferir pacientes tipo I y II, Tipo III casos excepcionales IV al VI nivel hospitalario
I) Paciente sano que requiere cirugía sin antecedente o patología agregada
II) Paciente que cursa con alguna enfermedad sistémica, pero controlada
III) Paciente que cursa con alguna enfermedad sistémica no controlada
IV) Paciente con enfermedad sistémica incapacitante
V) Paciente, se opere o no, tiene riesgo inminente de fallecer (24 hrs posteriores a valoración)
VI) Paciente con muerte encefálica, órganos a extirparse para trasplante
N.A: No aplica
